# Implementing a sepsis prediction score in out-of-hours primary care: Feasibility and acceptability study

**DOI:** 10.1080/13814788.2025.2574869

**Published:** 2025-11-10

**Authors:** Feike J. Loots, Lonneke A. van Vught, Minou van den Brande, Sophie Jepma, Bryce Renkema, Arthur R.H. van Zanten, Karin Kaasjager, Ann Van den Bruel, Johannes B. Reitsma, Kevin Jenniskens, Abeer Ahmad, Sibyl Anthierens, Roderick P. Venekamp

**Affiliations:** a Department of General Practice and Nursing Science, Julius Center for Health Sciences and Primary Care, University Medical Centre Utrecht, Utrecht University, Utrecht, The Netherlands; b Department of Intensive Care Medicine, Ziekenhuis Gelderse Vallei, Ede, The Netherlands; c Department of Human Nutrition and Health, Wageningen University and Research, Wageningen, The Netherlands; d Division of Acute Medicine, Department of Internal Medicine, University Medical Center Utrecht, Utrecht, The Netherlands; e Department of Public Health and Primary Care, KU Leuven, Leuven, Belgium; f Department of Epidemiology and Global Health, Julius Center for Health Sciences and Primary Care, University Medical Centre Utrecht, Utrecht University, Utrecht, The Netherlands; g Department of Family Medicine and Population Health, University of Antwerp, Antwerp, Belgium

**Keywords:** Sepsis, prediction score, out-of-hours general practitioner cooperative, feasibility, acceptability, implementation

## Abstract

**Background:**

Clinical scoring systems can help predict sepsis and guide treatment. We developed and validated a new sepsis prediction score for acutely ill adult patients visited at home by a general practitioner (GP) of an out-of-hours (OOH) cooperative.

**Aim:**

To assess the feasibility and acceptability of implementing this score during OOH home visits.

**Design and Setting:**

Theory-informed mixed-methods study at two OOH GP cooperatives in the Netherlands between January and June 2024.

**Method:**

GPs calculated the sepsis score in adult patients during home visits and completed a questionnaire about uptake and usability. Perspectives and experiences were explored through focus groups and semi-structured interviews.

**Results:**

106 GPs visited 271 patients at home, of whom 105 were judged acutely ill by the GP. The score’s uptake was 77% in all patients and 85% in those acutely ill. 91% of GPs rated the score as convenient to use, and 66% considered the score reliable and accurate. GPs reported that the score influenced their decision to refer the patient to the hospital in 6% (15/271; 11 referred, four not referred) of all cases and 10% (10/105, all referred) in acutely ill. GPs expressed that they did not solely rely on the score but found it helpful to raise sepsis awareness and as an adjunct to clinical decision-making.

**Conclusion:**

Implementing a new score for acutely ill adult patients visited at home during OOH primary care is feasible, and deemed acceptable and useful by GPs, however future research focusing on clinical validation and cost-effectiveness is needed.

## KEY MESSAGES

A newly developed sepsis score showed high uptake and acceptability among GPs during home visits.

The score supported clinical decision-making but was rarely used as the sole determinant for referral.

Further research is needed to evaluate the score’s clinical impact and cost-effectiveness in routine primary care.

## Introduction

Sepsis is a life-threatening organ dysfunction caused by a dysregulated host response to an infection [1]. The World Health Organisation (WHO) declared sepsis a global health crisis in 2017, as it affects more than 48 million annually, resulting in 11 million deaths [2].

Timely recognition of sepsis and initiation of adequate treatment are essential to prevent morbidity and mortality [1,3]. In hospital settings, clinical warning scores such as the quick Sepsis Related Organ Failure Assessment (qSOFA) [4] and the National Early Warning Score (NEWS) [5] are used to improve early detection. The qSOFA score is a simple count of three clinical signs but has limited sensitivity [6,7], whereas the NEWS is a more complex score, hampering the use by GPs. Furthermore, both scores include respiratory rate, which has limited accuracy and poor interobserver agreement [8] and is not routinely measured by GPs [9], limiting their suitability in primary care.

Recently, our group developed a simple score consisting of six variables (age > 65 years; temp > 38 °C; systolic blood pressure ≤ 110 mmHg; heart rate >110/min; peripheral oxygen saturation ≤ 95%; altered mental status), to predict sepsis (based on the SEPSIS-3 criteria 1] among acutely ill adults who were visited at home by a general practitioner (GP) of an out-of-hours (OOH) GP cooperative [10]. The score showed good calibration and discrimination in internal and external validation with predictions similar to the NEWS [10]. However, information about its usability and acceptability by GPs – key drivers of successful implementation in daily clinical practice – are lacking.

This study aimed to evaluate the feasibility and acceptability of routinely implementing the new sepsis scoring system during OOH GP home visits by employing a mixed-methods approach, integrating both quantitative and qualitative analyses.

## Methods

### Design and setting

This mixed-methods feasibility study consisted of two phases: a prospective implementation study (January 8 and March 31, 2024) followed by a qualitative investigation (April 23 and June 14, 2024). The qualitative component was guided by a theory-informed interview guide, drawing from implementation science frameworks and behavioural theories such as Consolidated Framework for Implementation Research (CFIR), Theory of Planned Behaviour and Health Belief model [11–13]. The study was conducted at two OOH GP cooperatives serving a combined suburban to rural population of nearly 400,000 inhabitants in the Utrecht region (Nieuwegein and Zeist), The Netherlands. In the Netherlands, OOH primary care is facilitated by GP cooperatives [14]. Patients can be seen in the OOH clinics, or during a home visit when indicated. Patients access a regional cooperative via phone for triage. The triage is performed by nurses using a computer-based decision-support triage system (the Netherlands Triage Standard). The triage nurses assess urgency, provide self-care advice, arrange physician visits, or call an ambulance, ensuring appropriate care. During a home visit, GPs are accompanied by a medically trained driver.

### Participants

Before the study was conducted, all GPs affiliated with the participating OOH GP cooperatives received a short communication by email on the importance of early sepsis recognition and an explanation of how to use and interpret the new sepsis score. During the study period, GPs received onsite training on the new sepsis score usage by a member of the research team before commencing their home visit shift and were instructed to measure and calculate the score during all home visits during physical examination except for those related to children, terminally ill and deceased patients. GPs were provided with pocket cards summarising the individual items and sepsis score-based risk categories (Table 1). GPs were informed about the risk of sepsis in each risk score group [10]. The ultimate decision to refer a patient to the hospital (or not), however, remained at the discretion of the GP.

**Table 1.** Sepsis prediction score of 6 variables, resulting in a score ranging between 0–6 points and subsequent advice per risk category on the score.

	Age > 65 years		1 point
	Tympanic temperature > 38 ⁰C		1 point
	Systolic blood pressure ≤ 110 mmHg		1 point
	Heart rate > 110 beats/minute		1 point
	Peripheral oxygen saturation ≤ 95%		1 point
	Altered mental status		1 point
0–1 points, low risk (3%)		No Emergency Department (ED) referral for suspected sepsis	
2–3 points, intermediate risk (29%)		Consider ED referral if there are signs of sepsis or there is diagnostic uncertainty	
4–6 points, high risk (76%)		Direct ED referral advised	

Accompanying drivers were also involved in the study. Drivers were instructed to remind GPs to calculate the sepsis score and to assist with vital sign measurements and score calculations. Their perspectives were included in the qualitative evaluation to capture insights on the feasibility and practicality of implementing the score in daily OOH workflows.

### Quantitative investigation: Uptake and usability

Following each home visit, GPs were asked to complete a paper case report form (CRF) containing anonymised background information on every home visit (patients’ age category, primary complaint), and whether the patient was acutely ill according to the judgement of the GP. Also, GPs were asked to answer the following questions for every home visit: i) Did you calculate the new sepsis score? ii) What was the score result? iii) Did you refer the patient to the hospital? and iv) Did the result of the score influence your referral decision? At the end of the shift, GPs were asked to rate eight usability and acceptability items on a 5-point Likert scale (Table 2).

**Table 2.** Summary of the GPs’ answers to the questions on the case report forms (*N* = 109).

	1: Strongly disagree N(%)	2: Disagree N(%)	3: Neutral N(%)	4: Agree N(%)	5: Strongly agree N(%)
The score was easily calculated during home visits	1(0.9)	1(0.9)	5(4.6)	47(43.1)	55(50.5)
I felt confident in the calculation of the score	0(0.0)	3(2.8)	9(8.3)	48(44.0)	49(45.0)
I felt confident in the interpretation of score results	0(0.0)	4(3.7)	30(27.5)	43(39.4)	32(29.4)
The score provided reliable and accurate results	0(0.0)	6(5.7)	34(32.1)	39(36.8)	27(25.5)
It felt safe using the score to inform referral decisions	5(4.9)	7(6.8)	62(60.2)	21(20.4)	8(7.8)
I have (partially) based management decisions based on score results	35(32.7)	36(33.6)	26(24.3)	10(9.3)	0(0.0)
The score led to increased confidence in deciding whether or not to refer a patient	24(23.1)	22(21.2)	44(42.3)	14(13.5)	0(0.0)
I intend to use the score in the future (beyond this study)	6(5.7)	11(10.4)	48(45.3)	35(33.0)	6(5.7)

### Qualitative investigation: Experiences and views

All GPs and drivers of the two OOH GP cooperatives were invited to participate in focus group discussions to explore their experiences and perspectives on the application of the new sepsis score using a semi-structured topic guide (Table S1). A semi-structured interview approach guided all interviews, allowing participants the flexibility to emphasise and expand on topics they deemed significant. Probing questions were employed as needed to clarify responses in more depth. All interviews were transcribed verbatim and continued until data saturation was achieved.

### Data analyses

The quantitative data from the CRFs were entered into a cloud-based data management system with an electronic data capture tool (Castor Electronic Data Capture). Descriptive statistics were performed using IBM SPSS Statistics, version 29.0.1.

Analysis of the qualitative data combined deductive and inductive methods to capture both anticipated and evolving themes with depth and nuance. Transcripts were imported into NVivo (release 14.23.1) to support data analysis. Initially, a deductive coding framework was applied, grounded in the key topics from the topic guide. This coding structure was then refined through inductive analysis, where repeated, open engagement with the data allowed new themes and insights to evolve.

To enhance the rigour and trustworthiness of the findings, researcher triangulation was applied. The multidisciplinary research team, comprising primary care, qualitative research, and implementation science experts, engaged collaboratively throughout the coding and interpretation process, bringing diverse perspectives to the analysis and challenging assumptions. Reflexivity was maintained by encouraging team members to critically reflect on how their own disciplinary backgrounds and potential biases might influence data interpretation.

## Results

### Participants

A total of 106 GPs completed 133 CRFs during the 12-week study period, containing data on 271 patients. The 133 CRFs are estimated to correspond to just below half of the total of 300 home visits shifts that have been performed during the study period were assigned. Of the patients, 42% were female, and 61% were aged over 75 years (Table 2). The most common reasons for home visits were respiratory complaints (26%), followed by gastrointestinal complaints and altered mental status (both 12%). In 39% (105/271), patients were judged acutely ill by the GP. In acutely ill patients, the most common entry complaints were respiratory complaints (39%), followed by fever (16%).

### Quantitative investigation: Uptake and usability

The new sepsis score was calculated in 77% (208/271) of all patients, 85% (89/105) of those who were judged to be acutely ill and in 72% (119/166) of those who were deemed not acutely ill (Table 3).

In the 208 patients for whom the sepsis score was measured, 32% (67/208) had a low, 50% (104/208) an intermediate and 18% (37/208) a high-risk category score (Table 3). The GPs referred 19% of the patients to the emergency department in the low-risk group, 32% in the intermediate, and 84% in the high-risk group (Table 3). GPs reported that in 6% (15/271) of patients, the score influenced the referral decision (11 referred, 4 not referred). The impact of the score on referral decisions was most prominent in high-risk patients (16%; 6/37 versus 7% (7/104) and 3% (2/67) in the intermediate and low-risk groups, respectively). In 10% (10/105) of those judged acutely ill, the score impacted the referral decision (all 10 referred) (data not shown).

**Table 3.** Distribution of the results of the new sepsis score per risk group for the total cohort (*n* = 208) and acutely ill patients (*n* = 90), stratified by referral status.

	Total			Acutely ill		
	Total	Referred		Total	Referred	
Score	*N* = 208	Yes *N* = 77	No *N* = 131	*N* = 89	Yes *N* = 55	No *N* = 34
Low risk (0–1 points), n (%)	67 (32)	13 (17)	54 (41)	16 (18)	6 (11)	10 (29)
Intermediate risk (2–3 points), n (%)	104 (50)	33 (43)	71 (54)	42 (47)	21 (38)	21 (62)
High risk (4–6 points), n (%)	37 (18)	31 (40)	6 (4.6)	31 (35)	28 (51)	3 (8.8)

**Table 4.** Baseline characteristics of patient contacts included in the quantitative analyses.

	Total	Sepsis score measured		Acutely ill	Sepsis score measured	
	(*n* = 271)	Yes (*n* = 208)	No (*n* = 63)	(*n* = 105)	Yes (*n* = 89)	No (*n* = 16)
Sex, n (%)						
Male	130 (48.0)	102 (49.0)	28 (44.4)	52 (49.5)	46 (51.7)	6 (37.5)
Female	141 (52.0)	106(51.0)	35 (55.6)	53 (50.5)	43 (48.3)	10 (62.5)
Age category, n (%)						
18–54 years	23 (8.8)	21 (10.4)	2 (3.3)	8 (7.8)	8 (9.1)	0 (0.0)
55–64 years	26 (9.9)	22 (10.9)	4 (6.7)	11 (10.7)	10 (11.4)	1 (6.7)
65–74 years	49 (18.7)	40 (19.8)	9 (15.0)	26 (25.2)	21 (23.9)	5 (33.3)
75–84 years	61 (23.3)	46 (22.3)	15 (26.7)	26 (25.2)	20 (22.7)	6 (40.0)
>85 years	103 (39.3)	74 (36.6)	29 (48.3)	32 (31.1)	29 (33.0)	3 (20.0)
Entry complaint*						
Respiratory	70 (26.2)	61 (29.6)	9 (14.8)	40 (38.8)	34 (38.6)	6 (40.0)
Fever	23 (8.6)	20 (9.7)	3 (4.9)	16 (15.5)	15 (17.0)	1 (6.7)
General malaise	23 (8.6)	20 (9.7)	3 (4.9)	8 (7.8)	8 (9.1)	0 (0.0)
Altered mental status	31 (11.6)	27 (13.1)	4 (6.6)	12 (11.7)	9 (10.2)	3 (20.0)
Gastrointestinal	31 (11.6)	23 (11.2)	8 (13.1)	10 (9.7)	10 (11.4)	0 (0.0)
Urogenital	8 (3.0)	4 (1.9)	4 (3.3)	2 (1.9)	1 (1.1)	1 (6.7)
Cardiac	11 (4.1)	9 (4.4)	2 (1.6)	5 (4.9)	5 (11.4)	0 (0.0)
Neurological	6 (2.2)	5 (2.4)	1 (1.6)	2 (1.9)	2 (2.3)	0 (0.0)
Trauma	27 (10.1)	18 (8.7)	9 (14.8)	3 (2.9)	2 (2.3)	1 (6.7)
Other	37 (13.9)	19 (9.2)	18 (29.5)	5 (4.9)	2 (2.3)	3 (20.0)

*4 missing values.

**Table 5.** Proportion of GP (strongly) agree or disagree on four relevant constructs based on the questions answered on the case report forms.

	1: Strongly disagree N (%)	2: Disagree N (%)	3: Neutral N (%)	4: Agree N (%)	5: Strongly agree N (%)
The score is convenient to use	0.5	1.8	6.4	44	48
The results of the score are trustworthy	1.6	5.3	40	32	21
Influence on referral decisions	28	27	33	11	0
Intent to use in future	5.7	10	45	33	5.7

Of the participating GPs, 93% (strongly) agreed with the question, ‘The score was easily calculated during home visits’ (Table 4). In comparison, 10% agreed with the question, ‘I have made management decisions based on the score results’. On the question ‘I intend to use the score in the future (beyond this study)’, 33% of the GPs agreed, 6% strongly agreed, while 10% disagreed and 6% strongly disagreed (Table 4). A summary of the questionnaire results by construct is shown in Table 5.

### Qualitative investigation: Experiences and views

Two focus group meetings were held, one consisting of 3 GPs and one consisting of 4 drivers. Five semi-structured interviews with individual GPs were conducted, given the logistical challenges of gathering more than 3 GPs per focus group session. Four main themes were identified: the perspectives on (i) diagnosing sepsis, (ii) practicality, (iii) reliability and (iv) future role and implementation of the score. A full list of interview quotes can be found in Table S2.

#### Theme I: Balancing clinical intuition with objective scoring in diagnosing sepsis

In diagnosing sepsis, GPs frequently rely on clinical intuition, often described as a ‘gut feeling.’ As one GP expressed, this intuitive approach is deeply ingrained in clinical practice: ‘As a GP, you usually work very intuitively’ (I2, GP). While GPs recognised the value of clinical intuition, they also emphasised the importance of objective sepsis scoring to support their initial suspicions. Beyond objective parameters, factors such as rapid symptom progression, concerns expressed by family members, and patient’s frailty could further raise GP’s suspicion of sepsis.

GPs also expressed that sepsis tends to be underdiagnosed in general practice: ‘We all know that it [sepsis] is missed a lot in general practice’ (I4, GP), emphasising the challenges in the timely and accurate diagnosis of sepsis in the outpatient setting.

#### Theme II: Practical usability and integration of the sepsis score

Both GPs and drivers expressed that the score’s design aligns well with existing practices, making it feasible for routine use. GPs valued the score’s simplicity and the inclusion of routinely available parameters: ‘What helps is that these are familiar signs and symptoms that we already collect anyway’ (I1, GP). Drivers echoed this positive assessment, noting that using the score did not increase their workload, an important factor in evaluating its practicality in time-sensitive, out-of-hours settings.

#### Theme III: Perceived reliability of the sepsis score

GPs generally felt that the sepsis score aligned with their initial clinical judgement or ‘gut feeling’. However, they emphasised the need for further clinical validation before the score could be widely adopted. Concerns were particularly raised about the score’s sensitivity, with some GPs feeling that it flagged patients as higher risk more frequently than anticipated based on their clinical assessment: ‘When you see those sepsis criteria, they’re super strict, I think. As in, you get there pretty quickly: if I then look purely at that score, I often found people on that score to be sicker than I found them in real life’ (FG2, GP). In line, drivers indicated that GPs tended to seek additional clinical justifications for admitting a patient rather than relying solely on a high sepsis score.

#### Theme IV: Future role and implementation of the sepsis score

GPs recognise that the primary hurdle for successful implementation lies in raising awareness of the score and motivating their colleagues to incorporate it into their routines: ‘Well, I know many of my colleagues won’t do that [incorporate], so finding uniformity in that is, I think, the biggest challenge’ (I2, GP).

GPs suggested that involving drivers more actively in future implementation efforts could enhance its uptake: ‘The driver […] can, of course, be included in such a score list, that driver can also calculate that himself’ (I3, GP).

GPs reported that they primarily use the score to complement their clinical intuition. Several GPs continued to use the score even after the study concluded, reflecting its potential value in supporting clinical decision-making and raising sepsis awareness.

## Discussion

This mixed-methods study investigated the feasibility and acceptability of implementing a new sepsis score during home visits of two OOH GP cooperatives in the Netherlands. It revealed that the score was commonly used during the study period. Overall, the score was measured in 77% of all patients and in 86% of those judged acutely ill by GPs.

The sepsis score was found easy to use, making it feasible for routine application in acutely ill adult patients visited at OOH primary care. Its simplicity and compatibility with current workflows were key factors contributing to its successful integration, as highlighted by both GPs and their accompanying drivers. This collaborative approach further enhanced the score’s practicality in the OOH setting.

GPs generally trusted the score’s results, finding its components aligned with their clinical reasoning. However, they noted that the score often indicated a higher sepsis risk than their initial clinical judgement suggested. In 7% of all patients and 10% of those identified acutely ill, the score influenced the decision to refer the patient. GPs emphasised that while the score rarely dictated referral decisions on its own, it served as a valuable tool to increase sepsis awareness and supported clinical decision-making as an adjunct to their judgement.

Most participating GPs expressed openness to using the score in the future. However, broader adoption would require further clinical validation studies and incorporation into national guidelines to ensure widespread support and confidence in its use.

To our knowledge, only one UK-based study reported data on the use of a sepsis score (NEWS) in primary care [15]. This study found that the NEWS score was calculated in 30% of patients admitted to the hospital with suspected sepsis by GP-support teams and in 70% by GPs [16]. Albeit differences in study populations hamper comparison, we found a high uptake of our sepsis score by GPs in a more general population of acutely ill adults visited at home during OOH primary care.

A key strength of the study is its mixed-method approach, integrating both quantitative and qualitative data, which provided comprehensive insights for future implementation of the sepsis score. Furthermore, the real-world OOH primary care setting ensures the findings are highly relevant for large-scale implementation efforts. Nevertheless, several limitations deserve further attention. First, since study participation was voluntary and GPs who completed the study forms may have been more inclined to use the sepsis score and since the participation to the qualitative part of the study was also voluntary, selection bias cannot be completely ruled out. This could have led to an overestimation of the uptake and acceptability of the score. Second, we included nearly all adults who were visited at home during OOH primary care, since sepsis may present with less typical symptoms in the early stages. Therefore, the usability and acceptability results may reflect more generic opinions about the sepsis score rather than its specific utility as a decision support tool in clinical scenarios of possible sepsis. Third, the qualitative evaluation of GPs’ experiences was based on a small sample, which may limit the depth and representativeness of the findings. Fourth, data on why GPs decided to refrain from calculating the sepsis score were not collected. Even though this was addressed during focus groups and semi-structured interview, for individual cases this remains unknown. Fifth, judgement on appropriateness of patient referral was beyond the scope of this study. Finally, the findings may not be directly transferable to other countries with different healthcare organisations.

Our study suggests that a new sepsis prediction score may be acceptable and feasible for use during out-of-hours home visits in primary care, at least under study conditions. However, the high uptake and positive evaluations may partly reflect the greater motivation of participating GPs during a research project. The score influenced hospital referral decisions in 7–10% of patients, underscoring its potential to enhance early sepsis identification in this setting. However, this study was not designed to evaluate the clinical and cost-effectiveness of routine use of the sepsis score, prior to widespread implementation of the sepsis score, further research is needed focusing on clinical validation and cost-effectiveness. Early referral of patients with sepsis could be expected to decrease hospital costs and improve patient outcomes, while referral of patients who do not need hospital treatment could increase healthcare costs and unnecessary use of healthcare resources. Based on the results of this study, the increase in unnecessary referrals is limited, but a collection of follow-up data, including patient outcomes and healthcare costs, is needed to confirm the cost-effectiveness.

## Conclusion

Implementing a new sepsis prediction score for acutely ill adult patients visited at home by OOH GPs is feasible, acceptable, and useful for practitioners. While the score shows promise in early sepsis recognition, further research is needed to establish its clinical and cost-effectiveness for routine use.

## Supplementary Material

Supplemental Material

## Data Availability

The data that support the findings of this study are available from the corresponding author upon reasonable request. Due to privacy and ethical considerations, data cannot be shared publicly.
