# Supplementary material for: Implementing a sepsis prediction score in out-of-hours primary care: Feasibility and acceptability study
Source: Eur J Gen Pract. 2025 Nov 10;31(1):2574869. doi: 10.1080/13814788.2025.2574869 (PMC12604108; doi:10.1080/13814788.2025.2574869)
Supplement: Supplemental Material [file IGEN_A_2574869_SM9400.docx]

**Supplementary Material for**

**Implementing a sepsis prediction score in out-of-hours primary care: feasibility and acceptability study**

Feike J. Loots, Lonneke A. van Vught, Minou van den Brande, Sophie Jepma, Bryce Renkema, Arthur R.H. van Zanten, Karin Kaasjager, Ann van den Bruel, Hans Reitsma, Kevin Jenniskens, Abeer Ahmad, Sibyl Anthierens, Roderick P. Venekamp.

|  | | **Page** |
| --- | --- | --- |
|  | |  |
|  | |  |
| **Table S1.** Semi-structured topic guide | | 2 |
| **Table S2.** Interview quotes | | 4 |
|  |  |  |
|  |  |  |
|  | |  |
|  | |  |
|  | |  |

**Supplementary Tables**

**Table S1**. Semi-structured interview topic guide

| **1. Relevance of the condition sepsis and the use of the sepsis score** |
| --- |
| Experiences with sepsis as general practitioners during home visits:   - How would you describe your overall experience dealing with patients potentially suffering from sepsis during home visits?   - Can you share specific examples of situations where you suspected sepsis during a home visit?   - What signs and symptoms do you primarily focus on when suspecting sepsis during home visits? - Follow-up steps after suspicion of sepsis: - What steps do you typically take when sepsis is suspected? - Are there specific follow-up steps you find more challenging to implement than others?   Relevance of the sepsis score (Awareness):   - How do you assess the suitability of the sepsis score compared to your previous clinical judgement of sepsis? - Does using the sepsis score prompt you to consider the diagnosis of sepsis more frequently? - How would you evaluate the overall usefulness of the sepsis score? |
| **2. General perceptions of using the sepsis score** |
| Experiences during home visits:   - How did you use the sepsis score? - In what way was the score presented to you? Was the information provided in the written information letter and verbal explanation by research member sufficient to clarify how to use the score?   - Information letter   - Verbal explanation - What has your experience been with using the sepsis score during home visits?   - Attitude   - Risk reduction   - Ease of use   - Learning curve - To what extent do you trust the score's results?   - Low score = no sepsis - High score = sepsis   Decision-making moments:   - At which moments or phases of the consultation, and for what reasons, did you decide to use the sepsis score?   - Can you provide a concrete example of this?   Decline in research form submissions:   - We observed that over the three months of the study, fewer research forms were completed.   - Is this something you also experienced?   - Do you think this is due to decreased use of the score or merely less frequent completion of research forms? |
| **3. Impact on clinical decision making** |
| Influence on clinical decision making:   - In what ways did the score’s results influence the decision to refer or not to refer a patient?   - Low score = no referral   - High score = referral   Adjustments during the study:   - Did your routine for assessing patients with possible sepsis change during the study? |
| **4. Intention to use after the study** |
| Practical usability in the future:   - Would you consider using this score yourself in the future?   - Why or why not? - How do you view the practical usability of the sepsis score moving forward?   - Use during home visits (as in the study’s setting)   - General use for all patients (consultations/home visits in your own practice)   Sharing insights and support needs:   - What can you share with each other about how this score could be used? - Can you tell us what support you would need to improve its implementation? - Can you identify what support others would likely require? |

**Table S2.** Interview quotes

| Theme | Subtheme | Quotations |
| --- | --- | --- |
| 1. Perspectives on  sepsis | 1.1 Intuitive  Approach and  Clinical Cues in  Sepsis Diagnosis | “As a GP, you usually work very intuitively” (I2, GP).  “Always of course fever, blood pressure, heart rate, overall... What does someone look like. Consciousness.” (FG 2, GP)  “… and the course of the illness, meaning how quickly it progresses, I always find important. That is not included in this score. And actually, I always consider a person's frailty as well. But that's not measurable, is it?” (I 2, GP) |
|  | 1.2 Daily Practice  Challenges and  Collaborative  Approach | "We all know that it [sepsis] is missed a lot in general practice" (I 4, GP)  “That's been my experience though [...] it can catch you off guard like that.” (FG 2, GP)  “I must say that those times I did see it, someone was also really seriously ill, [...] and then I did recognize it quickly. It is of course more difficult when you are still a bit in that preliminary stage, then you don't recognize it as quickly.” (I 5, GP)  “Am I not underestimating this situation? Because I think that's exactly [...] with those elderly people, that we do sometimes miss the earlier signs of sepsis. Yes, and then at the next visit, we find a critically ill patient.” (I 1, GP)  “And, it's kind of funny, because as a general practitioner you don't like to send somebody in just for the numbers, but you actually have to do that with sepsis.” (I 2, GP)  “I did experience sepsis several times and also thinking back I still sometimes think, didn't I miss something here in those years?" (FG2, GP) |
| 2. Practical use | 2.1 User-Friendly  Yet TimeConstrained  Implementation | “What helps is that these are generally parameters that we already collect anyway” (I1, GP).  “Especially If you do out-of-hours home visits of course, because then the driver does all these measurements. You check yourself if there is an altered mental state. You just have to fill in the score list in your head, and then you have it. So that does work very practically.” (I 4, GP)  “Because he [the score] was certainly simple.” (FG 2, GP)  “While you do have to learn to work with it, it's not a difficult list.” (I 3, GP)  “It's practical, yes. Yes very, yes. [Because] actually you know all those things. Then you just list it and give them a value.” (I 2, GP)  “No, it [the workload] was not perceived as shocking. And for us it’s… The measurements we do anyway.” (FG 1, driver)  “GPs, of course, do quite a lot with examinations and so on, so they are used to having to fill out lists often.” (FG 1, driver) |
|  | 2.2 Varied Timing of Score Utilization  and the Role of  Reminders | “And I must say that did change a bit over time, because when I first got it I thought, oh another list, what should we do with it? And at a certain point, I started using it and I thought: oh, this is actually quite useful to objectify it a bit more for yourself! Yes, so there was a turning point.” (I 3, GP)  “I filled it [the score] out after the home visit, but then I had already made my assessment during the consultation, like: what am I going to do? And then my assessment usually matched what was on the card.” (I 5, GP)  “Actually [using the score] is a checklist in your head whilst doing the physical examination, but at least before you go into the policy. So [...] before you determine your policy you determine, is there possibly sepsis?” (I 4, GP) |
| 3. Reliability score | 3.1 Perceived  Trustworthiness  and Sensitivity of  the Score | “When you see those sepsis criteria, they’re super strict, I think. As in, you get there pretty quickly: if I then look purely at that score, I often found people on that score to be sicker than I found them in real life” (FG2, GP)  “If you have studied that such a score is a reliable predictor of sepsis... Then I would think: okay, it really becomes worth using that score.” (I 1, GP)  “And I was confident that it was also reliable. A few times I did both, but with the SIRS criteria you have to count the respiratory rate. Well, I don't feel like doing that and it's always a bit unreliable. It was the best we had, and not very good. And that's why I started using that score list and it's extraordinarily nice.” (I 4, GP)  “Often they would look for one more thing, just to make sure they didn't refer for nothing. Otherwise, in the hospital, you get a name of [...] doctor-I-refer-anything.” (FG 1, driver) |
|  | 3.2 Heightened  Sepsis Awareness  and Influence on  Safety-Netting | “But I think the score helps you to have it a bit sharper in the differential diagnosis and maybe give a bit sharper safety-net. With any change, deterioration, don't wait until 8 a.m. the next morning, but also raise the alarm at night, I think.” (FG 2, GP)  “It supports your clinical judgment, so it makes your own thoughts more concrete. […] But, I don't quite dare say that [the score changed my policy].” (I 4, GP)  “We just did our job and then filled in that score afterwards. But of course that also meant that in those shifts we actually did not let that score guide our policy at all.” (I 1, GP)  “Yes indeed, it [the score] didn't influence it [policy]. No, I didn't calculate it, say, during the visitation, but then afterwards I would fill it out in the car and see what the score was.” (I 5, GP) |
| 4. Future  perspectives | 4.1 Fostering  Awareness and  Facilitating Usage | "Well, I know a lot of my colleagues won't do that, so finding uniformity in that is, I think, the biggest challenge" (I2, GP).  “Well look, of course any GP, or any person, is hard to change. First of all, he has to see the usefulness and necessity of it. So well, usefulness is: it's easier, you can do it with whatever you have in your bag. So that's how you can entice.” (I 4, GP)  “If you keep repeating... the power of repetition, because at the beginning you always have resistance, but the tenth time you see the list you think: oh, maybe I should look at it anyway. [...] And what also helps is indeed trying to point out the added value.” (I 3, GP)  “But that, at least for me, is something [...] I can always find, that I don't have to look up a link again in an email somewhere, but that you just know: it’s right here.” (FG 2, GP) |
|  | 4.2 Perceived Value  and Potential  Applications of the  Score | "The driver [...] can, of course, be included in such a score list, that driver can also calculate that himself" (I3, GP).  “I know that many general practitioners are not very good at it. [...] Ultimately, the clinical judgment is still the most important; everyone relies on that. But for me, working with this just makes it a bit more certain. And even though it is not yet validated, I am willing to use this tool to have a bit more ammunition.” (I 4, GP)  “Yes, what might be a nice application is if you integrate it into the ABCD criteria. For example, if you completely fill out the ABCD criteria, which you should ideally do for every sick patient, and if there is also a fever, a sort of sepsis recommendation rolls out.” (I 5, GP) |
| Abbreviations: FG focus group interview, I individual interview, GP general practitioner | | |
